# The Role of Ultrasound for the Personalized Botulinum Toxin Treatment of Cervical Dystonia

**DOI:** 10.3390/toxins13050365

**Published:** 2021-05-20

**Authors:** Urban M. Fietzek, Devavrat Nene, Axel Schramm, Silke Appel-Cresswell, Zuzana Košutzká, Uwe Walter, Jörg Wissel, Steffen Berweck, Sylvain Chouinard, Tobias Bäumer

**Affiliations:** 1 Department of Neurology, Ludwig-Maximilians-University, 81377 Munich, Germany; 2 Department of Neurology and Clinical Neurophysiology, Schön Klinik München Schwabing, 80804 Munich, Germany; 3 Djavad Mowafaghian Centre for Brain Health, Division of Neurology, University of British Columbia Vancouver, Vancouver, BC V6T 1Z3, Canada; devavrat.nene@ubc.ca (D.N.); silke.cresswell@ubc.ca (S.A.-C.); 4 NeuroPraxis Fürth, 90762 Fürth, Germany; praxis@neuropraxis-fuerth.de; 5 2nd Department of Neurology, Comenius University, 83305 Bratislava, Slovakia; zuzanakosutzka@gmail.com; 6 Department of Neurology, University of Rostock, 18147 Rostock, Germany; uwe.walter@med.uni-rostock.de; 7 Neurorehabilitation, Vivantes Klinikum Spandau, 13585 Berlin, Germany; joerg.wissel@vivantes.de; 8 Department of Paediatric Neurology, Ludwig-Maximilians-University, 80337 Munich, Germany; sberweck@schoen-klinik.de; 9 Schön Klinik Vogtareuth, 83569 Vogtareuth, Germany; 10 Centre hospitalier de l’Université de Montréal, Montréal, QC H2X 3E4, Canada; Sylvain.chouinard.med@ssss.gouv.qc.ca; 11 Institute of Systems Motor Science, University of Lübeck, 23562 Lübeck, Germany

**Keywords:** botulinum toxin, cervical dystonia, ultrasound

## Abstract

The visualization of the human body has frequently been groundbreaking in medicine. In the last few years, the use of ultrasound (US) imaging has become a well-established procedure for botulinum toxin therapy in people with cervical dystonia (CD). It is now undisputed among experts that some of the most relevant muscles in this indication can be safely injected under visual US guidance. This review will explore the method from basic technical considerations, current evidence to conceptual developments of the phenomenology of cervical dystonia. We will review the implications of introducing US to our understanding of muscle function and anatomy of common cervical dystonic patterns. We suggest a flow chart for the use of US to achieve a personalized treatment of people with CD. Thus, we hope to contribute a resource that is useful in clinical practice and that stimulates the ongoing development of this valuable technique.

## 1. Introduction

Ever since a number of high-quality clinical trials have shown excellent safety and superior efficacy, precisely performed and dose-adequate intramuscular injections of botulinum neurotoxin serotype A (BoNT/A) have been the first-line treatment for cervical dystonia (CD), without any equally effective alternative [1].

From the beginning of the BoNT/A therapy in CD in the 1980s, these injections have been executed by movement disorder experts who have been trained to be highly familiar with the anatomy of the human cervical region, even in the case of dystonic alterations of muscle size and head position. There is a shared belief in the community that an experienced injector will know, from own or adopted experience, which muscles to choose for treatment, and that he/she is able to inject those muscles with sufficient precision using anatomical landmarks, or in specific situations, using electrophysiological support to detect pathological electromyography (EMG) activity, guiding the needle to the best location for deploying the toxin, e.g., [2,3,4]. However, experience arises from expectations and on the basis of the currently circulating models about the disease. This is also and especially true for CD. It is worth having a look at those models.

For the stratification of his treatment approach, Tsui et al. (1986) suggested to differentiate the clinical patterns of CD as if the cervical spine would represent a singular joint, i.e., torticollis, antecollis, retrocollis, laterocollis [5]. While such a reduced construction of CD has its merits in terms of simplicity and clarity, other phenomenological varieties were not represented. Ultimately, certain muscles involved in CD, such as the small head muscles, were not identified as targets for BoNT-treatment.

Visualizing the muscles of the neck by examining cervical computed tomographies, Gerhard Reichel and his team developed a novel classification system for CD that he termed the ColCap-concept, to encompass the large variety of clinical patterns he had observed [6,7]. They suggested that the complex cervical spine with its seven vertebrae could be reduced to two main functional units. First, the head movements in the occipito-atlanto-axial articulation, and second, the lower cervical spine from C3 to C7. He termed the former “caput”, and the latter “collis” movements. Using various visualization techniques, ultrasound (US) being one of them, a team of neurologists and radiologists had similarly demonstrated the obliquus capitis inferior muscle to be a relevant contributing muscle for CD [8].

Even before this work on CD, pediatric neurologists, neurologists, and anesthesiologists had already initiated the use of US guidance for BoNT injection for children with cerebral palsy [9,10], and for plexus blockade [11]. Their work described key features of the technique, such as technical requirements, anatomical aspects, and distinguishing the target from contour lines or surrounding structures. Thus, US guidance became the standard procedure for BoNT injections in children with cerebral palsy [12], and for injecting local anesthetics into the vicinity of neural structures [13].

This scoping review will forcefully argue for an US based approach to rely less on preconceived models of CD but to assess the individual dystonic pattern of the patient, and for US guidance for BoNT injections to become routine use, as we have done in previous publications [14,15]. We suggest using US-guided EMG for complex patterns of CD, as only such a combined approach achieves to match precise topographical with pathognomonic functional information that is necessary to characterize the individual dystonic pattern. To this end, we introduce the reader to the application of US by raising awareness of the technique’s potential, reviewing key technical details and relevant aspects of neck anatomy, illustrating explanations with state-of-the-art images of the muscles of the neck, and providing an overview of the current sparse scientific literature. We suggest a stepwise workflow to visualize the role of US for the personalized treatment of CD, and report four illustrative clinical cases.

## 2. Technical Background

US is a well-established imaging tool in muscular diagnostics [16]. The US systems suitable for muscle identification and botulinum toxin injection guidance are essentially the same as those used for vascular diagnostics of the vessels of the neck and extremities. In principle, all modern devices can be used. A linear array transducer is required for undistorted image acquisition. When choosing the US transducer frequencies, there is a trade-off between the optimal resolution versus increased depth penetration. This results from the physical reality that higher frequency US signals providing higher resolution images are increasingly attenuated with deeper tissue penetration. On the other hand, lower frequency signals providing lower resolution images achieve a better penetration of deeper tissues. Modern harmonic imaging techniques have helped to overcome this inherent limitation by detecting harmonic resonance frequencies in higher frequency ranges, thus allowing for higher resolution images at greater depths. In general, the optimal resolution for superficially seated target muscles is achieved with frequency ranges of 12–18 MHz, and for deeper seated target muscles with lower frequency ranges of 3–10 MHz. For the purposes of muscle and needle imaging during injections with BoNT/A, outstanding image quality is not a prerequisite. Most modern US systems will provide multi-frequency transducers (e.g., 4–15 Hz) that can sufficiently image deep-seated muscles, and will also cover higher frequencies for more superficial regions with better resolution. Please note that a 27 gauge injection needle will be detected in an off-plane approach mostly from the motions of surrounding tissues upon insertion of the needle rather than by direct visualization of the needle.

## 3. Ultrasound Improves Anatomical Knowledge

It might seem obvious but cannot be overstated that the use of US to inject BoNT/A in people with CD improves the topographical and functional anatomical knowledge of the practitioner in a substantial way. This newly acquired knowledge interacts with the clinical evaluation of the patient, with the conception of the injection protocol, and, ultimately with the outcome of the treatment. The following aspects of US anatomy seem noteworthy.

### 3.1. Layers and Compartments

**Figure 1:**
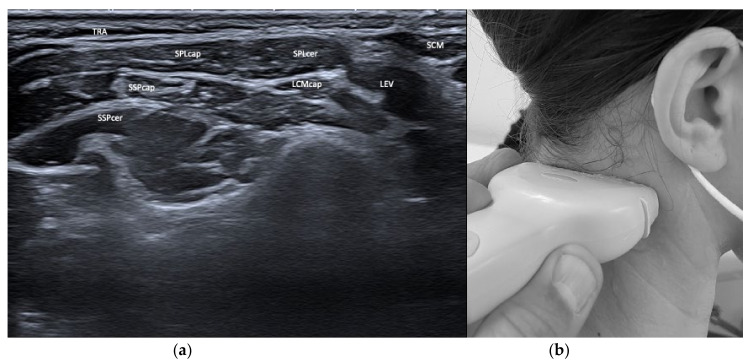
(**a**) US image of the muscles of the neck at the level of C4. Depicted are trapezius (TRA), splenius capitis (SPLcap), semispinalis capitis (SSPcap) and cervicis (SSPcer), longissimus capitis (LCMcap), levator scapulae (LEV), and sternocleidomastoideus (SCM); (**b**) location of the sonar probe.

**Figure 2:**
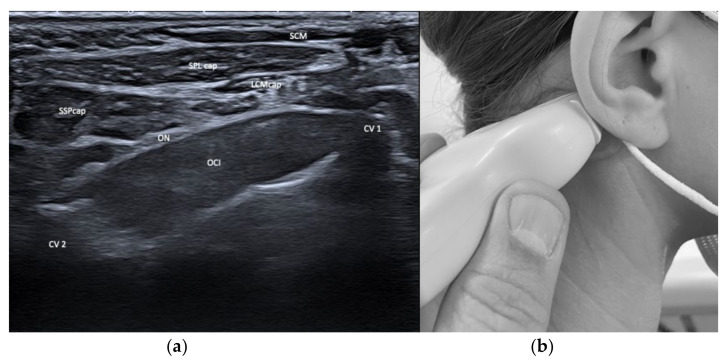
(**a**) US image of the muscles of the neck at the level of C1 (atlas, CV1) and C2 (axis, CV2). Depicted are sternocleidomastoideus (SCM), splenius capitis (SPLcap), semispinalis capitis (SSPcap), longissimus capitis (LCMcap), and obliquus capitis inferior (OCI), as well as the greater occipital nerve (ON); (**b**) Location of the sonar probe.

From a posterior approach, we differentiate two compartments that are relevant for BoNT/A treatment of CD: (i) The superficial compartment containing trapezius (TRA), splenius capitis and cervicis (SPL), longissimus (LCM), semispinalis capitis and cervicis (SSP), and levator scapulae (LEV), and (ii) the deep compartment containing the small head muscles obliquus capitis superior and inferior (OCS, OCI) and the rectus capitis major and minor (RCMj, RCMi). Below the C2 level in the deep compartment, we locate the erector spinae muscle containing various longitudinal systems which are rarely relevant for the treatment of CD. The differentiation of various depths can easily be achieved by US visualization, but not by using external landmarks alone, such as the mastoid. Additionally, see Figure 1 and Figure 2.

**Figure 3:**
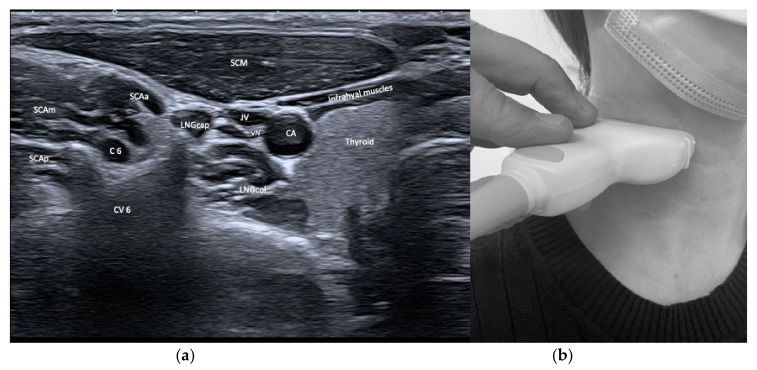
(**a**) US image of the muscles of the anterior neck at the level C6. Depicted are sternocleidomastoideus (SCM), scalenus anterior (SCAa), medius (SCAm) and posterior (SCAp), longus colli (LNGcol), longus capitis (LNGcap), and infrahyoid muscles. Carotid artery (CA), jugular vein (JV), vagal nerve (VN), thyroid gland, cervical vertebra (CV6), and spinal nerve C6 can be visualized, too; (**b**) location of the sonar probe.

**Figure 4:**
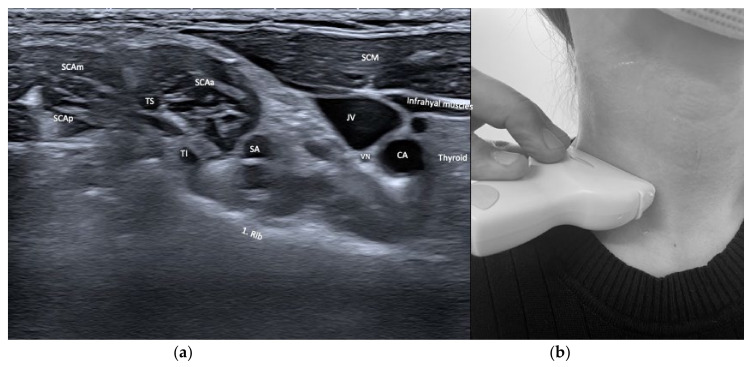
(**a**) US image of the muscles of the anterior neck at the level of the first rib. Depicted are sternocleidomastoideus (SCM), scalenus anterior (SCAa), medius (SCAm) and posterior (SCAp), and infrahyoid muscles. Carotid artery (CA, subclavian artery (SA), the Truncus superior (TS), Truncus inferior (TI) of the brachial plexus, the jugular vein (JV), thyroid gland, vagal nerve (VN), and the first rib can be visualized, too; (**b**) location of the sonar probe.

From laterally and anteriorly, the sternocleidomastoideus (SCM), the scaleni (SCA), and the infra- and suprahyoid muscles are depicted. The SCM shows relevant variety with its two origins from the clavicle and the manubrium, but is mostly injected close to its mastoid insertion. After years of repeated injections with BoNT/A, the SCM can become atrophic so it may be necessary to move the injection site caudally to inject muscular tissue. The scaleni muscles are in a delicate environment and in close vicinity to the brachial plexus and the pulmonal cupula. As their muscle bulk lies caudally, careful injection planning is required. See Figure 3 and Figure 4.

**Figure 5:**
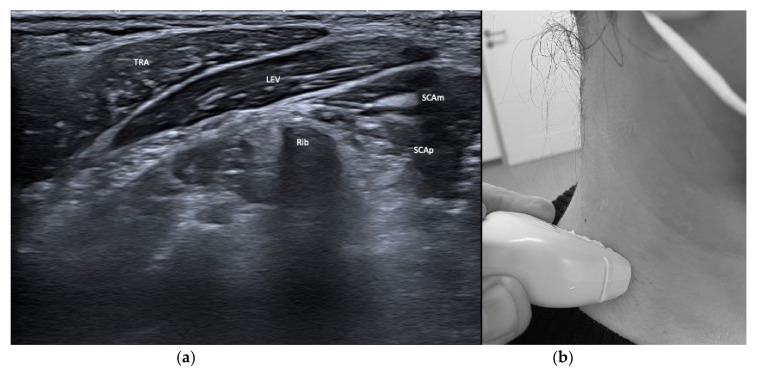
(**a**) US image of the lateral and lower muscles of the neck. Depicted are trapezius (TRA), levator scapulae (LEV), scalenus medius (SCAm) and posterior (SCAp). The rib can be easily identified from its sonar shadow; (**b**) location of the sonar probe.

From laterally, the levator scapulae’s (LEV) origins from vertebrae C1 to C3(4) can be traced to its insertion at the scapula. See Figure 5. The LEV gives a fine example for a variant phenomenology while observing dystonic activity within the same muscle. Depending on the punctum fixum, a dystonic LEV can produce laterotilt of head and neck or elevation of the shoulder. The phenomenology depends on what other muscles are involved in the pattern.

### 3.2. Orientation of Layers—Reciprocal Function of Neighboring Structures

In order to appreciate the need for precise injections of BoNT/A to neck muscles, it is highly relevant to understand the biomechanical architecture of cervical muscular orientation. Unlike muscles of the extremities, the cervical muscles do not form a parallel array but are layered in sheets, each only a few millimeters thick. Moreover, every layer is oriented in somewhat perpendicular orientation to the next layer. This criss-cross orientation stabilizes the neck, but at the same time, requires the injector to precisely choose the correct depth for the injections, as directly neighboring structures can have opposing functions, e.g., trapezius produces contraversion, splenius ipsiversion, and semispinales’ role for head turning is variously described, but they are the strongest extensors of the neck. Additionally, see Table 1. This precision in the range of millimeters is only achievable with real-time visual guidance as provided by US.

### 3.3. Biomechanical Basic Assumptions: Cross-Section and Lever Arm

Determining the relevant muscle pattern in CD should account for the two biomechanical components of muscle torque in any affected articulation: the muscle cross-section (bulk) and the lever arm, i.e., the distance from the center of articulation, which, for movements of the neck, are the various articulations of the cervical spine. Both components correlate linearly to the torque. Several conclusions with functional relevance may be drawn from these basic theoretical concepts and visualized with US imaging. First, SPL and SCM, both originating from the mastoid process, have the largest leverage, and both can have large cross-sections, making them the most important rotators of the head or neck. Second, among the small head muscles, the OCI has the largest cross-section, making it most relevant for isolated rotation of the head. Third, rather tiny muscles, such as the platysma or infrahyoid muscles, can exert relevant torque on the vertebral column as they have large leverage.

### 3.4. Safety Issues and Imaging of Relevant Neighboring Structures

US imaging is intrinsically safe, and a pain and radiation free procedure. In addition, US imaging allows the direct visualization of the neurovascular structures, such as the vertebral and carotid arteries, the jugular vein, the spinal nerves, the vagal nerve, the brachial plexus, and other peripheral nerves such as the greater occipital nerve, which can thus be avoided during the injection process. This safety feature hands US guidance an undisputable advantage over other guiding techniques, such as the EMG, and, of course, non-guidance. Indeed, only the use of US has made it possible to research the accuracy of injections, which for US guided injections in CD, was estimated to be between 81–100% [17,18,19]. Furthermore, it is also proposed that as long as low volume injections are performed at a slow pace (to avoid backflow) and into the region with greatest width, the toxin would remain localized to the target muscle [18]. It is therefore conceivable that given its high accuracy, US would be associated with less spread of toxin to the surrounding structures and in turn with fewer adverse events. There is, however, conflicting evidence about the impact of US on occurrence of adverse events such as dysphagia in the very limited available literature. A small open label study, comparing EMG and US concluded that addition of US to EMG completely eliminated dysphagia [20]. On the contrary, a retrospective chart review conducted over a period of one year on 75 patients with CD observed no improvement in dysphagia on using US guidance [21]. It should be kept in mind, however, that the evidence from these studies is of low quality and precludes drawing any firm conclusions. Even though the evidence is limited, it is only logical to assume that, because of greater accuracy and direct visualization of neuromuscular structures and muscle anatomy, injections performed under US are safer than unguided techniques.

### 3.5. Learning Anatomy Anew—From Clinical Assumptions to Visual Feedback

As we have outlined above, injection schedules for CD are often based on preconceived disease models that delineate typical patterns, sometimes using algorithmic flowcharts to achieve better personalization [22]. While those frameworks for CD have achieved proven results, the authors can report from daily experience that many people with CD do not fit the established categories. CD is a highly individual motor syndrome, and while it is tempting to simplify our treatment regimen, a personalized treatment approach that is aligned with actual topographic and functional anatomical findings in the individual holds the promise to deliver a satisfactory treatment outcome, including for those patients that do not respond to standard regimen. Thereby, only the regular use of the US for simple and complex cases will lead to the procedural abilities and the intuitive knowledge of the anatomical details that make this technique useful, especially for the complex cases.

Treating people with CD requires a profound understanding of the physiological sensory-motor function, and of the pathological distortions specific to the dystonic condition. Seemingly dystonic alterations may result from paresis, gravity, bony deformity, or fibrosis [23]. The US provides the means to uncover the etiology of these differential diagnoses. Recently, a deep-learning algorithm has been applied to US images of neck muscles obtained in CD patients and controls. The authors describe distinct subtypes of CD based on ultrasonographic patterns of neck muscle shape [24]. US based real-time shear wave elastography has been demonstrated to identify the most hypertensive muscles in patients with CD [25]. US combined with innovative image analysis tools may enhance the individual assessment and planning of therapy for CD patients in future settings. These studies demonstrate that the technology continues to evolve and may offer even more benefits in the future.

**Figure 6:**
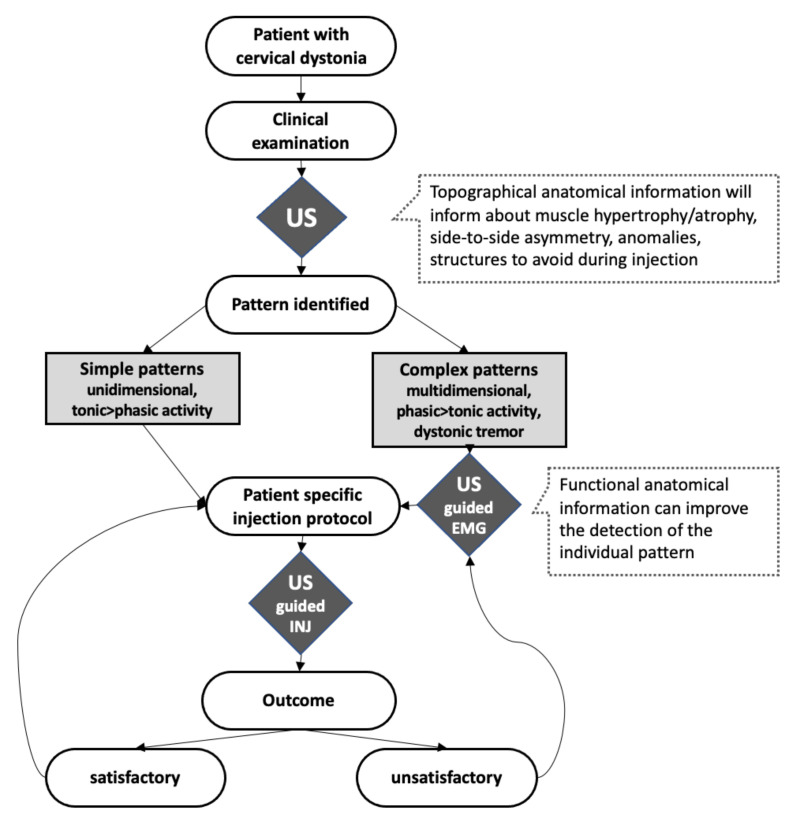
Presentation of a workflow which the authors propose for the use of US and EMG for a personalized BoNT/A treatment of people with CD. In addition to the obvious role of US and EMG for injection guidance, the importance of US for learning the patient’s individual anatomy and dystonic pattern is pointed out. Therefore, the authors recommend the regular use of US even for people with CD who present with seemingly uncomplicated patterns.

Taken together, there is accumulating evidence that the visualization of the dystonic syndrome of CD will lead to a novel conception of the disease that does not search for similarities and typical patterns, but for personalized patterns. We have charted a clinical workflow that might help establish a management loop for more satisfactory treatment outcomes in people with CD. See Figure 6.

### 3.6. Injecting BoNT/A Using US Guidance

Typically, BoNT/A is injected with an out-of-plane injection approach, which involves inserting the needle along the short axis of the transducer, almost perpendicularly to the skin surface, reaching the target location with a short and most direct trajectory [26,27]. The main disadvantage of this technique is that the needle is visualized in the cross-section as a hyperechoic dot. On the other hand, when using an in-plane approach, the needle is inserted along the long axis of the transducer, which often entails a longer route to the target location. Although the in-plane approach allows for the visualization of the entire needle trajectory, it requires very precise needle maneuvering, which is often difficult to achieve, especially in patients with CD.

Irrespective of the technique, US imaging of the injected muscle provides the examiner with a unique opportunity of observing the spread of the fluid along the direction of the muscle fibers [28]. It is intuitive to think that the thicker the needle size, the better would be its visibility on US imaging, owing to greater reflection of the US beam. Thus, if better visual guidance is desired, the injector can use a thicker needle size of 0.5/25 or 0.6/23 mm/gauge, while thinner needles of 0.3/30 or 0.4/27 mm/gauge are mainly used to minimize the pain during the procedure.

Injecting BoNT/A close to the motor endplate seems desirable [29,30]. However, further studies are needed to determine its practical application in patients with CD as there is insufficient knowledge about the precise distribution of motor endplates in the majority of the cervical muscles.

### 3.7. The Relevant Muscles for CD

Potentially all muscles can be relevant for people suffering from CD. However, the protocols of the large RCTs and the label status for the available BoNT/A preparations do not mention many of them. Fortunately, using US, we can demonstrate their relevance for the disease, as is shown in the patient case in Section 6.1. Table 1 lists most, but not all muscles, that are involved in CD, and provides information regarding the need for US imaging and safety issues. We also comment on further clinical aspects.

## 4. Combining US with the EMG

**Figure 7:**
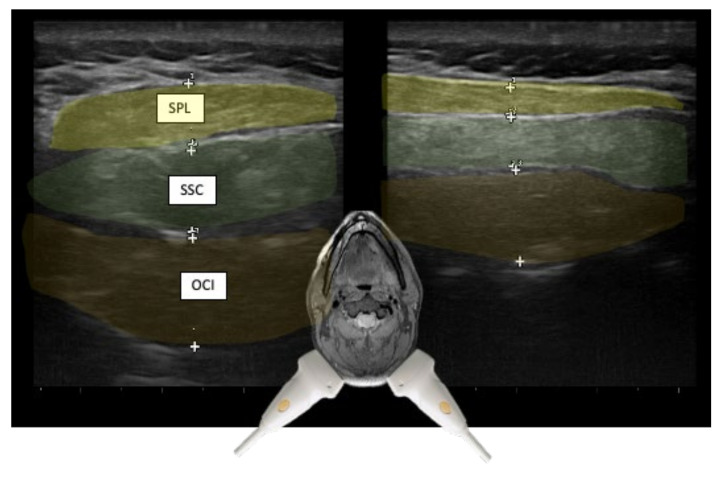
US images from posteriorly in a cross-sectional plan from a de novo patient. The comparison of left and right sides reveals prominent asymmetry of muscle thickness and, consequently, depth for injection. Splenius capitis (SPL; yellow), semispinalis capitis (SSC; green), and obliquus capitis inferior (OCI, orange).

EMG as a guidance technique for botulinum toxin injections enables targeted placement of the toxin in muscle areas where most activity is detected from the EMG signal. Accordingly, at least two prospective randomized controlled studies showed better treatment outcomes when EMG guidance was compared to injections without the use of guidance [31,32]. However, the method has two disadvantages: First, without the use of specialized EMG techniques (e.g., spectral analysis), it is not possible to distinguish dystonic activity from compensatory activity [33,34]. This might lead to erroneous injections in antagonistic muscles, as the muscles in the neck region, some of which have the opposite function, lie very close to one another in layers only a few millimeters thick [35]. Additionally, see Figure 1, Figure 2, and Figure 7. It is simply impossible to unequivocally assign the detected EMG activity to a specific muscle in every case. Second, EMG needle insertions are performed blindly, and the examiner receives no information about structures he/she needs to avoid, such as vessels or nerves. Both disadvantages can be avoided by combining the *functional* guidance technique EMG with US as an *anatomical* guidance technique. Using such a combined approach, the activity in dystonic no-no-tremor was located, in most examined cases, in the deep-seated obliquus capitis inferior muscles, and not in the commonly injected splenius capitis muscle [35]. A practical application for the combined use of both methods would be an US-guided mapping of EMG activity for the individual patient with CD. The mapping may set out with the most commonly injected muscles, and additional muscles based on clinical evaluation are added to the procedure, at the discretion of the examiner. Based on the results, an injection protocol will be created and could be applied for subsequent injections. In case of an insufficient response or the occurrence of adverse events, the authors suggest to repeat the evaluation. We have visualized this approach in Figure 6. Such a combined approach might not be necessary for patients with simple patterns, such as unidimensional rotation or tilt patterns. The authors suggest combining EMG and US for patients with complex patterns that vary in many spatial dimensions, or show phasic dystonic activity, and for patients with dystonic head tremor.

A recent large survey found dystonic tremor to occur in up to 58% of patients [36]. Dystonic tremor correlates with duration and severity of dystonia. It is often not recognized, or misdiagnosed. In the experience of the authors, head tremor in many patients is associated with a high disease burden, if standard injection protocols targeting muscles such as splenius or sternocleidomastoideus fail, and tremor-causing deep muscles such as the OCI, which require US guidance for injection, are not included in the treatment plan.

## 5. Evidence for the Use of US for Botulinum Toxin Treatment for CD

To date, there is an unfortunate paucity of research on the use of US for CD. The following studies have applied US in this indication (see Table 2). Taken together, there is a substantial lack of high-level evidence that promotes or refutes the use of US for the injection of BoNT/A above other guidance techniques.

## 6. Clinical Pearls

To exemplify our hypotheses, we present four cases from our daily routine where US imaging played a pivotal role for the detection of a personalized pattern, and a successful treatment.

### 6.1. “Depth Matters”—Do Not Underestimate the 3rd Dimension

The visualization of the muscles to be injected with US has further diagnostic advantages besides the control of the needle position. Some of these are listed below. In patients with CD, there is often a significant asymmetry of the thickness of cervical muscles due to the dystonic activity [7,41]. See Figure 7.

### 6.2. “Expect the Unexpected”—Variant Anatomy

**Figure 8:**
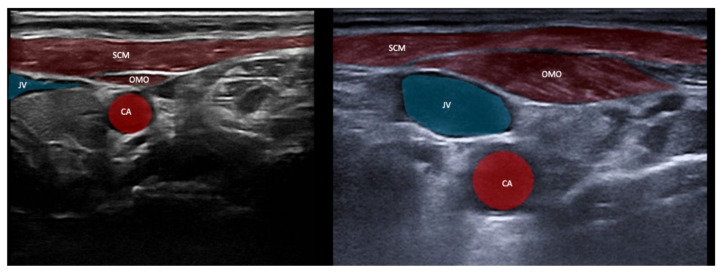
Illustration of the sternocleidomastoid (SCM) and omohyoid (OMO) muscles in two patients with CD. Please note the different depths of the two images (left 3 cm, right 2 cm). The close proximity of the SCM and OMO to the carotid artery (CA) and the jugular vein (JV) is clearly visible. Likewise, the markedly different muscle bulk of the OMO with obvious hypertrophy is evident on the right.

While randomized controlled trials have to standardize procedures to produce data for labeling the investigated medical product, the individual patient often does not fit into the constructs of the disease models. See Figure 8 for an example.

### 6.3. “Watch Out for Tremor”

Another diagnostic aspect of the visualization of muscles with US is the involvement of the OCI in dystonic head tremor. Even if tremor activity of the OCI is not always as clearly visible as in Video S1, such a finding has high therapeutic relevance, as one can recognize immediately that this muscle is involved in a no-no tremor.

See Supplementary Video S1 that demonstrates a short video snippet with a tremulous OCI.

### 6.4. “It May Not be Dystonia”

Another diagnostic aspect is the differentiation of dystonia from other causes of torticollis. In children and adolescents, focal dystonia of the neck is an absolute rarity and, for example, fibrosis of the SCM with a shortening and corresponding malposition of the head is far more common.

**Figure 9:**
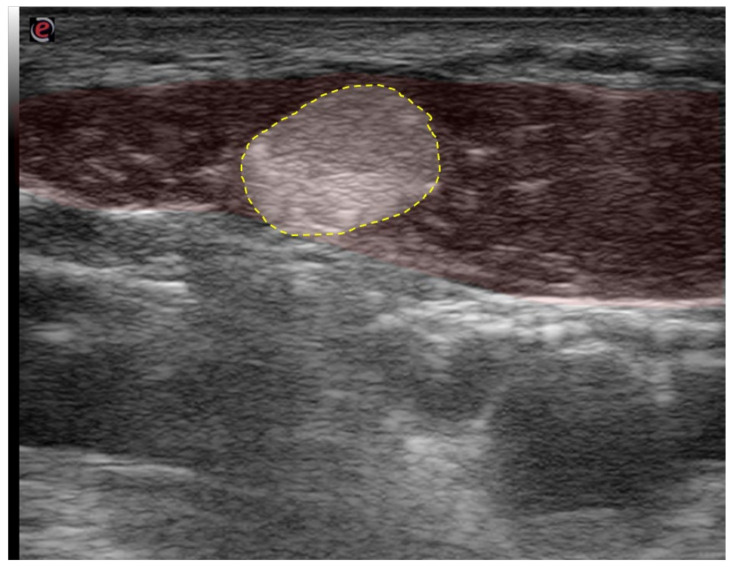
US image of a fibrotic cord in the SCM in a young female patient with torticollis. The SCM is shaded red, fibrotic cord is outlined with a yellow dotted line.

Figure 9 illustrates the case of a 16-year-old patient who presented with a newly acquired torticollis following a traumatic sports incident. She had been hit by a handball thrown to the neck. Using US, the diagnosis of a fibrotic cord was made. The cord was subsequently surgically removed, and the patient was symptom-free thereafter.

## 7. Conclusions

US is an increasingly widely available, non-invasive, pain free, radiation-free, cost-effective, and time-saving guidance technique. Dedicated randomized controlled research assessing objective clinical outcome parameters comparing the different guidance techniques are sparse, and we strongly recommend to push for more research in this domain. We expect that the use of US in clinical trials will contribute to better standardization and thus higher quality of research. In the absence of definitive research trials into the use of US guidance, the authors’ opinion based on their combined and extensive clinical experience and limited existing literature can be summarized as follows:

- US offers the advantage of unambiguously identifying the targets for BoNT/A treatment of CD.
- US allows the anatomically precise injection even into deep-seated muscles.
- US increases the reproducibility of therapy, and thus, the efficacy and safety of long-term BoNT/A treatment of people with CD.
- Treating with US improves the anatomical knowledge—topographically and functionally—of the practitioner, leading to more individual injection protocols.
- Combining US with EMG guidance opens the path to the analysis of complex clinical patterns of CD that often are unresponsive to standard protocols.

## Figures and Tables

**Table 1:** Muscles of the neck, their predominant functions, and the relevance of US for BoNT/A treatment. +, ++, +++ signifies relevant, very relevant and most relevant, respectively.

Muscle (Abbreviation)	Predominant Function from Neutral Position	Relevance of US		Neighboring Structures	Comment
		For Localization	To Avoid Side Effects		
Infra-/suprahyoid	anteflexion of head and neck	+++	+++	Thyroid gland	Dysphagia
Sternocleido-mastoid (SCM)	Contraversion of head and neck Ipsitilt of head and neck Anteflexion of head and neck with bilateral activation	+	++	Infrahyoid/supra-hyoid muscles, omohyoid, carotid artery, jugular vein	Dysphagia (particularly if injected bilaterally)
Longus capitis (LNGcap)	Anteflexion of head and neck	+++	+++	Carotid artery, jugular vein, vagus nerve, phrenic nerve	Authors recommend a transoral injection
Longus colli (LNGco)	Anteflexion of neck	+++	+++	Carotid artery, jugular vein, vagus nerve, phrenic nerve	Authors recommend a transoral injection
Scalenus anterior (SCAa)	Anteflexion of neck	+++	+++	Thyroid, carotid artery, brachial plexus, phrenic nerve, lung	
Scalenus medius/posterior (SCAmp)	Ipsitilt of neck	++	++	Brachial plexus, lung	
Semispinalis capitis (SSPcap)	Extension of head	+	+	SPLcap, OCI	Strongest extensor muscle of head and neck
Semispinalis cervicis (SSPcer)	Extension of neck	+	+	SPLcer, TRA	
Splenius capitis (SPLcap)	Ipsiversion of head	++	++	major occipital nerve, SPLcap/cer, LSMcap, OCI	Prominent reduction in bulk from repeated injections possible
	Ipsitilt of head				
Splenius cervicis (SPLcer)	Ipsiversion of neck	+++	++	LEV, TRA, Longissimus cervicis	Relevant for full turn of the neck
	Ipsitilt of the neck				
Longissimus capitis (LCM)	Ipsiversion of head and neck	+++	++	SPLcap, SSPcap	Obligatory USG
Trapezius (TRA)	Extension of neck	+	+	LEV, Supraspinatus	
	Contraversion of neck				
Levator scapulae (LEV)	Lift of scapula	+	+	SPLcer, TRA	
	Ipsitilt of head and neck				Role for antecollis variants is discussed variably
Obliquus capitis inferior (OCI)	Ipsiversion of head	+++	+++	SSPcap, RCM, vertebral artery, greater occipital nerve	Adjacent muscles are all extensors of the head
Rectus capitis major (RCM)	Extension of head	+++	+++	SSPcap, OCI	

**Table 2:** Published research on the use of US for the treatment of CD with BoNT/A. The N corresponds to the number of participants.

Author	Year	*N*	Type of Study	Method	Main Result
Lee et al. [8]	2008	6	Retrospective chart review	US, CT, EMG, and SPECT	OCI is a relevant muscle in CD.
Hong et al. [20]	2012	5	Retrospective chart review	US and EMG	Dysphagia was eliminated using combined US and EMG guided injection.
Fujimoto et al. [37]	2012	1	Case report	US	For guiding injection into LNGcol US is recommended rather than EMG.
Huang et al. [38]	2015	105	Prospective, randomized, controlled	US combined with joint brace	Significant improvement in dystonia at 1, 3, and 6 months of treatment compared to non-guided injection
Allison et al. [39]	2016	1	Case report	US and EMG	Injection of LongCol was efficient in the improvement of antecollis.
Schramm et al. [35]	2017	35	Prospective, non-controlled	US and EMG	Muscle activity of OCI is present in tremulous CD and the injection of OCI using US is recommended.
Walter et al. [26]	2018	5	Retrospective chart review	US	Add-on US guided injections into the OCI led to better outcomes, especially of the tremulous component.
Tyslerowicz et al. [40]	2019	1	Case report	US and EMG	Injection of LongCol is recommended using EMG and US.
Kutschenko et al. [21]	2020	117	Retrospective chart review	US	US guided application failed to prevent dysphagia (retrospective study)

## Data Availability

Not applicable.

## Supplementary Materials

The following are available online at https://www.mdpi.com/article/10.3390/toxins13050365/s1, Video S1: Tremulous activity in the OCI.
